# Minimal Concentrations of Deoxynivalenol Reduce Cytokine Production in Individual Lymphocyte Populations in Pigs

**DOI:** 10.3390/toxins12030190

**Published:** 2020-03-18

**Authors:** Karolina Hlavová, Hana Štěpánová, Kamil Šťastný, Lenka Levá, Nikola Hodkovicová, Monika Vícenová, Ján Matiašovic, Martin Faldyna

**Affiliations:** Veterinary Research Institute, Hudcova 296/70, 62100 Brno, Czech Republic

**Keywords:** deoxynivalenol, pig, subclinical dose, lymphocytes, PBMC, cytokines, animal health, immunotoxicity

## Abstract

Deoxynivalenol (DON) is a mycotoxin frequently found in cereals, and pigs are one of the most sensitive farm species to DON. The aim of this study was to determine the effects of DON in very low doses on peripheral blood mononuclear cells (PBMC) and on particular lymphocyte subpopulations. The cells were exposed to 1, 10 and 100 ng/mL of DON and lymphocyte viability, proliferation, and cytokine (Interleukin (IL)-1β, IL-2, IL-8, IL-17, Interferon (IFN) γ and tumor necrosis factor (TNF) α production were studied. Cells exposed to DON for 5 days in concentrations of 1 and 10 ng/mL showed higher viability compared to control cells. After 18 h of DON (100 ng/mL) exposure, a significantly lower proliferation after mitogen stimulation was observed. In contrast, an increase of spontaneous proliferation induced by DON (100 ng/mL) was detected. After DON exposure, the expression of cytokine genes decreased, with the exception of IL-1β and IL-8, which increased after 18 h exposure to 100 ng/mL of DON. Among lymphocyte subpopulations, helper T-cells and γδ T-cells exhibiting lower production of IL-17, IFNγ and TNFα were most affected by DON exposure (10 ng/mL). These findings show that subclinical doses of DON lead to changes in immune response.

## 1. Introduction

The toxin deoxynivalenol (DON), formerly named vomitoxin, belongs to the group B of trichothecene mycotoxins produced by Fusarium molds (e.g., Fusarium graminearum). DON is a tetracyclic sesquiterpenoid which contains a keto group at carbon 8 of the parent epoxytrichothecene nucleus. It is detectable as a contaminant of cereal grains such as wheat, barley and corn [1]; thereby it is the most frequently occurring mycotoxin in cereal-based food and feed in Europe and America [2]. DON is of high importance in farm animal feeding because of its frequent occurrence in cereal grains and its toxic effects at low doses [3,4]. Compared to polygastric animals and birds, which have a high bacterial content in the initial parts of their gastrointestinal tract, swine appear to be considerably more vulnerable to DON exposure. There is a high percentage of cereals in the porcine diet and pigs lack a rumen with microbiota with which to degrade mycotoxins before reaching the small intestine, where absorption of mycotoxins takes place [5,6,7]. Acute exposure to high levels of DON causes vomiting in pigs [8]. The effects of prolonged dietary exposure to DON are decreased weight gain and anorexia, leading to decreased slaughter weight and reduced feed efficiency [9].

The effects of DON are immunostimulatory or immunosuppressive depending on the dose and exposure frequency [10]. At the cellular level, DON targets the mononuclear cells of the immune system, stimulating pro-inflammatory gene expression at low concentrations, but at higher concentrations eliciting apoptosis of leukocytes that approach complete translational arrest [11,12]. The proliferation of lymphocytes can be reduced to 50% in the presence of 216 ng/mL of DON as observed in human cells [13]. The activity of T and B cells, including antibody production, is reduced and the percentage of lymphocyte population CD4^+^CD8^−^ present in the blood increases [4,14].

The immunomodulatory effects of DON are believed to be mediated through the ribotoxic shock response (RSR), primarily via the activation of kinases associated with ribosomes, a primary cellular target of DON [15]. RSR is responsible for the phosphorylation of mitogen-activated protein kinases (MAPK), ERK1, ERK2, JNK, and p38 [15]. Activated MAPKs stimulate the expression of mRNA and alter the levels of Interleukin (IL) -2, IL-4, IL-5, IL-6, IL-8 and Interferon (IFN) γ cytokine secretion [13,16,17,18,19].

The above-mentioned effects were observed in cells treated with DON in concentrations of 100 ng/mL and above. However, the concentration of DON in the serum of pigs after consumption of contaminated feed is usually much lower. Piglets fed a DON-contaminated diet with concentrations under the limit recommended by the EU (0.9 mg/kg) [20] exhibited DON serum concentrations 1–10 ng/mL [21,22]. Even though the oral route is the most frequent way of mycotoxin entry into the organism, it is very complicated to study the cellular impact of very small doses of mycotoxin via nutritional studies. There was no evidence that feeding DON in subchronic doses influenced the metabolic activity of porcine peripheral blood mononuclear cells (PBMC) or cytokine production [23]. Additionally, there are other ways mycotoxins can enter into the body, e.g., transplacental transmission from pregnant sows to fetuses [24,25], where a nutritional study would not be appropriate. In this case, an in vitro study would be a better method of determining immunotoxic effects, as the actions of the mycotoxin can be mimicked in a more controlled and even more detailed way. For this reason, the aim of this study was to investigate the effect of low concentrations of DON (1, 10 and 100 ng of DON per ml) on porcine PBMC and on lymphocyte subpopulations. The concentrations were chosen as an approximate range present in serum after DON-contaminated feed intake [26,27,28]. We focused on immune cell characteristics such as viability, ability to proliferate and cytokine production.

## 2. Results

### 2.1. Viability of PBMC after DON Exposure

The viability of PBMC (% of viable cells ± SD) after 18 h cultivation without the addition of DON (control) and treatment with 1, 10 and 100 ng/mL of DON was 89.0 ± 2.7; 88.7 ± 2.3; 88.5 ± 2.3 and 89.2 ± 2.5, respectively, and no statistically significant differences were found between the DON-treated and control cells. After 5 days of this treatment, the percentage of viable cells (±SD) was 53.9% ± 12.0%; 62.5% ± 12.9%; 61.0% ± 13.2% and 55.7% ± 10.5%, respectively. Significantly higher (*p* ≤ 0.01) percentages of viable cells were detected after treatment with 1 and 10 ng/mL DON compared to the control. These results indicate that short-term exposure to DON does not affect the viability of PBMC and very low concentrations of DON (1 and 10 ng/mL) can slightly increase the viability of cells during longer term cultivation.

### 2.2. PBMC Proliferation Activity after DON Exposure

The effect of DON exposure in concentrations of 1, 10 and 100 ng/mL on the responsiveness of lymphocytes to mitogen stimulation was studied. Mitogen-driven proliferation assay revealed that only the highest concentration used had a significant impact on lymphocyte proliferation. After 18 h exposure, stimulation indices (ratios between counts per minute (CPM) of mitogen-stimulated cells and CPM of non-stimulated cells) of samples treated with ConcanavalinA (ConA) and Pokeweed mitogen (PWM) were significantly lower compared to control cells. After 5 days of cultivation, a significantly lower stimulation index after 100 ng/mL DON exposure with PWM stimulation was found (Table 1).

Furthermore, spontaneous lymphocyte proliferation after DON exposure without mitogen stimulation was studied. A significant increase of proliferative activity after 18 h exposure to 100 ng/mL DON was observed. After 5 days of exposure, no significant differences in spontaneous lymphocyte proliferation were found between the DON-exposed and control cells (Table 2).

To conclude, exposure to DON in very low concentrations affects neither spontaneous nor mitogen stimulated proliferative responses of PBMC. Short-term exposure to 100 ng/mL DON decreases ConA and PWM induced proliferation. Long-term exposure does not have any significant effects on both mitogen-induced and spontaneous proliferation.

### 2.3. The Expression of mRNA Encoding Cytokine Genes in PBMC and Lymphocyte Subpopulations after DON Exposure

The expression of IL-1β, IL-2, IL-8, IL-17, tumor necrosis factor (TNF) α and IFNγ cytokines was determined in PBMC after 18 h and 5 days of DON exposure in concentrations 1, 10 and 100 ng/mL. The data are summarized in Table 3.

Our data show that all used concentrations of DON had a strong effect on cytokine expression. The expression of IL-1β and IL-8 increased after 18 h of DON (100 ng/mL) exposure. A significant decrease in the mRNA expression of IL-2, IL-17, IFNγ and TNFα after short-term DON exposure (10 ng/mL) was observed. Similarly, short-term exposure to 1 ng/mL DON decreased the expression of IL-2, IL-17 and TNFα. Long-term (5 days) exposure to DON significantly decreased the expression of nearly all of the observed cytokines in all concentrations, with the exception of IL-8 and TNFα after cultivation with 100 ng/mL, IFNγ with 10 ng/mL and IL-2 with 1 ng/mL, which were not affected.

In conclusion, PBMC exposure to DON leads mostly to a decrease in cytokine expression with the exception of IL-1β and IL-8. The mRNA expression of these cytokines increases after short-term exposure to the highest concentration of DON used.

**Figure 1:**
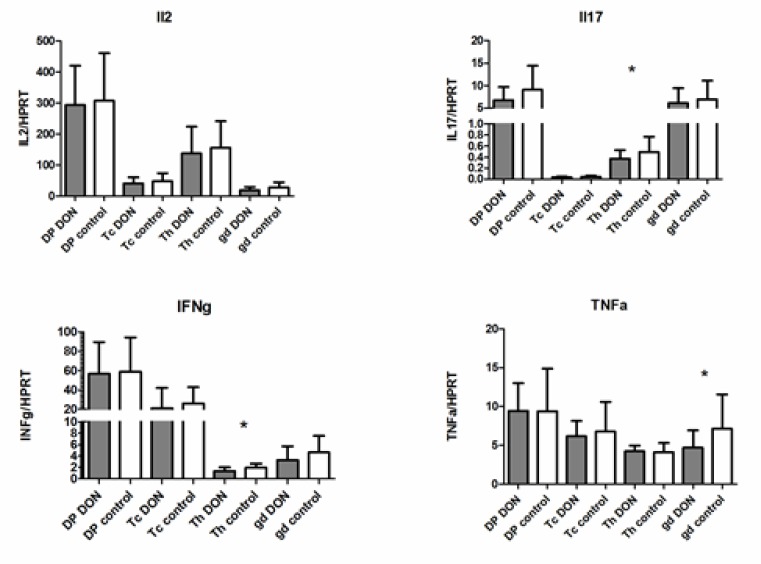
Expression of cytokine (IL-2, IL-17, IFNγ and TNFα) mRNA in lymphocyte subpopulations and the impact of DON exposure (10 ng/mL; 18 h). Values are expressed as mean of multiples of the reference gene expression ± standard deviation. Data of samples are from six pigs in biological duplicate. Statistically significant differences between DON exposed samples and control samples are marked with asterisk (* *p* ≤ 0.05). Tc—Cytotoxic T cells (CD3+γδTcR-CD4-CD8+), gd—γδ T cells (CD3+γδTcR+CD4-CD8+/-), Th—helper T cells (CD3+γδTcR- CD4+CD8-) and DP—double positive T cells (CD3+γδTcR-CD4+CD8+).

In accordance with qRT-PCR results of PBMC, which revealed that lymphocytic cytokine expression is mainly affected after exposure to low concentrations of DON, cytokine expression was measured in four T-lymphocyte subpopulations. Cytotoxic T cells (Tc; CD3+γδTcR-CD4-CD8+), γδ T cells (γδ; CD3+γδTcR+CD4-CD8+/-), helper T cells (Th; CD3+γδTcR-CD4+CD8-) and double positive T cells (DP; CD3+γδTcR-CD4+CD8+) were isolated with fluorescence-activated cell sorting (FACS), exposed to DON (10 ng/mL) for 18 h and then cytokine (IL-2, IL-17, IFNγ and TNFα) mRNA expression was determined with qRT-PCR. A slight decrease, but statistically insignificant, in the expression of all cytokines was observed in all subpopulations (Figure 1). Th cells exhibited a significant decrease inIL-17 and IFNγ expression, and a significant decrease inTNFα expression was observed in γδ. None of the studied T-lymphocyte subpopulations exhibited any significant response to DON exposure on IL-2 production.

### 2.4. Cytokine Production at the Protein Level in Lymphocyte Subpopulations after DON Exposure

**Figure 2:**
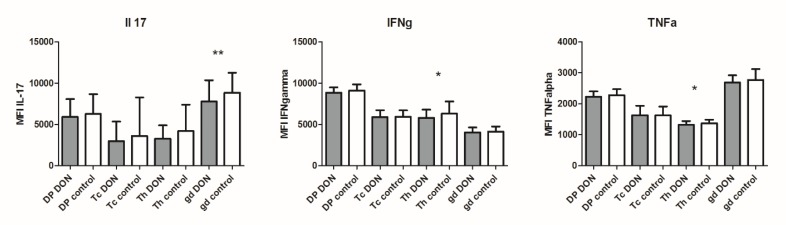
Production of cytokines (IL-17, IFNγ and TNFα) at the protein level in lymphocyte subpopulations and the impact of DON exposure (10 ng/mL; 18 h). The level of cytokine production is expressed as median of fluorescence intensity (MFI) for each lymphocyte subpopulation. Data of samples are from 12 pigs. Statistically significant differences between DON exposed samples and control samples are marked with asterisk (* *p* ≤ 0.05, ** *p* ≤ 0.01). Tc—Cytotoxic T cells (CD3+γδTcR-CD4-CD8+), gd—γδ T cells (CD3+γδTcR+CD4-CD8+/-), Th—helper T cells (CD3+γδTcR- CD4+CD8-) and DP—double positive T cells (CD3+γδTcR-CD4+CD8+).

To determine if the DON exposure effect on cytokine gene expression also occurs at the protein level, a flow cytometric analysis was used. An intracellular staining of cytokines with simultaneous staining of cell surface markers to detect cytokine production by individual lymphocyte subpopulations was used. In accordance with the results from qRT-PCR, there was a notably lower production of all studied cytokines in all subpopulations after DON exposure (10 ng/mL, 18 h) compared to control cells. A significantly lower production of IFNγ and TNFα was found in Th cells, and IL-17 production was significantly lower in γδ cells (Figure 2).

## 3. Discussion

The aim of this study was to investigate the effect of DON in concentrations comparable to those appearing in pig serum in real conditions after the ingestion of moderately contaminated feed (up to 0.9 mg/kg of feedstuff) according to the European Commission Recommendation [20]. The effect of such concentrations on immune cells has not been studied yet. The basic cellular parameter when studying the impact of mycotoxins is viability. It is known that high concentrations (≥3 μg/mL) of DON dramatically increase the apoptosis of porcine peripheral blood lymphocytes [29], in the spleen and lymph nodes [30]. In this study, although no significant impact of DON on PBMC viability after short-term treatment (18 h) was found, surprisingly, after a long-term culture (5 days), there was a significantly higher PBMC viability seen in 1 and 10 ng/mL DON treatment. These results are in accordance with Marin et al. [29], who observed this phenomenon after just 48 h of DON treatment in concentrations up to 300 ng/mL. This intriguing effect of low doses of DON on porcine PBMC has not been investigated fully and further studies, mainly on signal transduction and gene expression, are needed.

Apart from viability, changes in the ability to proliferate are a fundamental indication of toxic effects on cells. In the case of DON, these effects are strongly dependent on the concentration. High levels of DON (≥100 ng) inhibit proliferation in mouse lymphocytes [16] as with human and rat lymphocytes [13,31]; whereas in low concentrations, the effects can be disparate [32,33]. In this study, we examined the effect of DON on proliferation. PMBC were exposed to DON itself and to DON in combination with three commonly used lymphocyte stimulating mitogens (ConA, phytohemaglutinin (PHA) and PWM). ConA and PHA are selective T cell mitogens. They bind and cross-link components of the T cell receptor, and their ability to activate T cells is dependent on expression of the T cell receptor. PWM activates both T and B cells [34]. In our study, only the highest DON concentration used (100 ng/mL) had a significant impact on lymphocyte proliferation. Interestingly, in this study, was found that, when exposed to DON, the proliferation is inhibited if the cells are stimulated with ConA or PWM, similarly to the findings in mouse [19] and in porcine [33,35] ConA stimulated lymphocytes. On the contrary, a significant increase in blastogenesis and subsequent proliferation after DON exposure without mitogen stimulation was observed. A similar increase in blastogenesis and subsequent proliferation was observed in stimulated human, rat, mouse and also porcine lymphocytes when cultured in low (up to 100 ng/mL) concentrations of DON [29,36]. Distinct effect of DON on proliferation of stimulated and non-stimulated cells can be explained by possible block of the signaling [37] from T cell antigen receptors [38,39], whereas on its own DON stimulates the proliferation perhaps activating ERK kinases [40].

In this study, the effect of DON on cytokine production was studied both at the mRNA expression level and at the protein production level. The expression of the following cytokines was studied: IL-1β and IL-8 produced by activated monocytes/macrophages; IL-2, IL-17 and IFNγ produced by various subpopulations of activated lymphocytes; and TNFα produced by both lymphocytes and monocytes [41]. The effect of various DON concentrations on cytokine gene mRNA expression was studied in PBMC. A long-term (5 days) cultivation with DON led to a decrease in the expression of nearly all studied cytokines (IL-1β, IL-2, IL-8, IL-17, IFNγ, TNFα). This decrease should not be referred to cellular viability, which is positively affected by DON (as described above). Nevertheless, many authors described cytokine expression decrease in various tissues related to DON-contaminated feed consumption [32,42,43]. Unfortunately, these studies lack any information about the levels of DON in the observed tissues. In contrast, Meky et al. [13] documented an increase in IL-2 and IL-4 production and a slight inhibition of IL-6 production after 200 ng/mLDON exposurein human lymphocytes. Furthermore, Ouyang et al. [16] reported that DON at a concentration of 40 ng/mLincreases the production of IL-2, IL-5 and IL-4 in mouse CD4+ cells. In this study, wefound that short-term stimulation with DON at a concentration of 100 ng/mLincreased the production of IL-1β and IL-8. Both of these cytokines can be considered as pro-inflammatory and in peripheral blood are mostly produced bymonocytesin response to TLR, activated complement components or stimulation with other cytokines [44,45]. Increased levels of IL-1β and IL-8 may be part of the mechanisms causing the development and persistence of inflammatory diseases related to DON exposure [46,47]. Notably, on the other hand, short-term exposure to lower concentrations (10 and 1 ng/mL) of DON, did not affect the expression of IL-1β and IL-8, while the expression of IL-2, IL-17, IFNγ and TNFα decreased. A similar decrease in cytokine production in porcine spleen cells after DON-contaminated food ingestion was also described by Grenier et al. [48] and in accordance with these results, we focused on lymphocytes, as they are the major source of these cytokines [49,50,51,52]. The impact of DON exposure on T-lymphocyte subpopulations was studied at the levels of cytokine gene expression and the production of the cytokine as a protein. Four main T-lymphocyte subpopulations—Tc, γδ, Th and DP—were studied. These subpopulations represent the majority of porcine T cells [53]. Among all studied subpopulations, a significant effect of DON exposure was observed only in Th and γδ T cells. In all cases, we documented the decreased gene expression/protein production of IL-17, IFNγ and TNFα. It is known that long-term exposure to DON does not lead to any significant changes in the percentage of lymphocyte subpopulations [35,54,55]. Nevertheless, it is well documented that the above-mentioned cytokines play an important role in the immune response to antigens and the diminution of their levels leads to a decreased response to immunization [56,57,58,59,60]. A decreased response in the form of lower levels of specific antibodies in connection to DON exposure has also been described [48] and is associated with lower cytokine levels similar to our results. If the decrease in cytokine production is caused simply by translational arrest, or by the specific activation of signaling proteins [7], then it requires particularized molecular research. To confirm the effect of altered cytokine levels caused by DON exposure on the response to vaccination or infection, an extensive study using a very sensitive detection of DON levels in blood serum, detection of cytokine levels along with antibody and cellular response evaluation would be very useful. These results should serve as a cornerstone to reevaluate the limits of mycotoxins in porcine feed.

## 4. Conclusions

The impact of in vitro DON exposure on PBMC viability, proliferation, as well as cytokine production in PBMC and lymphocyte subpopulations was studied. We can conclude that low (100 ng/mL) DON concentrations have an effect on PBMC proliferation, increase the production of monocyte/macrophage cytokines (IL-1β, IL-8), decrease the production of cytokines by T-lymphocytes (IFN-γ, TNFα, IL-17, IL-2) after 18 h exposure, and significantly reduce cytokine expression after long-term exposure. Very low levels of DON (1 and 10 ng/mL) extend the PBMC viability after 5 days of cultivation. Nevertheless, the production of cytokines decreased after both long- and short-term DON exposure. These findings should be taken into account when animals are exposed to low-contaminated feed without having any clinical signs of mycotoxin intoxication but the immune response can be affected.

## 5. Materials and Methods

### 5.1. Animals and Blood Samples

Peripheral blood was collected by the external jugular vein puncture from twelve conventional, clinically healthy Large White pigs of both sexes, at the age of 3–6 months, kept at the Veterinary Research Institute experimental stables, 3 per pen and their feed was checked for DON absence [25]. The animal care and use protocol was approved by the Branch Commission for Animal Welfare of the Ministry of Agriculture of the Czech Republic (reference number MZE1214, approved 16 August 2016).Blood samples were heparinized with sodium heparin (Zentiva, Prague, Czech Republic, 25 IU/mL) and immediately processed.

### 5.2. Isolation of Peripheral Blood Mononuclear Cells

PBMC were isolated by gradient centrifugation on Histopaque 1077 (Sigma-Aldrich, St. Louis, MO, USA). Blood samples were diluted 1:2 in Dulbecco’s Phosphate Buffered Saline (DPBS; Lonza, Basel, Switzerland), layered onto Histopaque 1077 density gradient medium and centrifuged for 40 min at 626× *g*. Cells collected from the interface between the Histopaque medium and the sample were washed twice with DPBS and finally resuspended in RPMI 1640 medium supplemented with 10% foetal calf serum (FCS) (Gibco, Waltham, MA, USA), 100,000 IU/L penicillin, 100 mg/L streptomycin (both Sigma-Aldrich, St. Louis, MO, USA)) in concentration 1 × 10^6^ cells/mL.

### 5.3. Isolation of Lymphocyte Subpopulations with FACS

**Figure A1:**
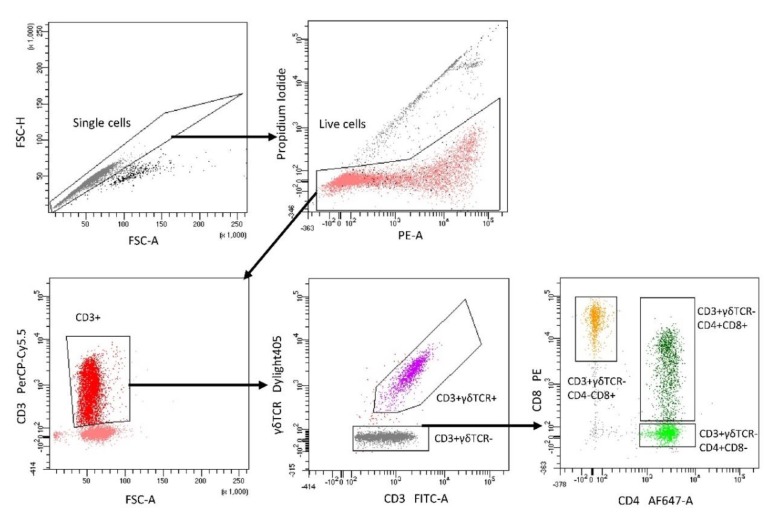
Gating strategy—Isolation of lymphocyte subpopulations with fluorescence-activated cell sorting (FACS). Doublets and dead cells identified by propidium iodide staining were excluded. Subsequently, T cells subpopulations were defined from all CD3 positive cells. Dot plots show data from one representative sample.

Four lymphocyte subpopulations were separated by FACS. Isolated PBMC were stained for surface markers CD3/CD4/CD8/γδTCR. The following combinations of primary antibodies were used: CD3 (FITC-conjugated, PPT3, Bio Rad, Hercules, CA, USA), CD4 (unconjugated, 10.2 H2, IgG2b, WSU, Pullman, WA, USA), γδTCR (unconjugated, PGBL22A, IgG1, WSU, Pullman, WA, USA) and CD8α (unconjugated, 76-2-11, IgG2a, WSU, Pullman, WA, USA). Fluorochrome-conjugated mouse isotype-specific goat antisera were used for unconjugated primary antibody visualization: Alexa Fluor 647 (anti-IgG2b, Invitrogen, Carlsbad, CA, USA), Dylight405 (anti-IgG1, GeneTex, Irvine, CA, USA), Phycoerythrin (anti-IgG2a, Invitrogen, Carlsbad, CA, USA). Dead cells were identified by propidium iodide staining and doublets were excluded from sorting. Tc, γδ, Th and DP (Figure A1) were sorted on BD FACS Aria Fusion operated by Diva software (BD Biosciences, Franklin Lakes, NJ, USA). Sorting was performed under the following conditions: nozzle size 70 µm, sheath pressure 70 psi, frequency 87 kHz and four-way purity sort mask. Sorted cells were collected into 5 mL tubes containing RPMI-1640 medium (Sigma-Aldrich, St. Louis, MO, USA) supplemented with antibiotics and 10% FCS. The tubes were centrifuged and the cells were resuspended in fresh medium. A small aliquot from each sample was subjected to immediate purity analysis. The average purities of the sorted cells were Tc 99.7%, γδ 97.3%, Th 98.1% and DP 98.2%.

### 5.4. Viability Assay

PBMC were seeded in 96-well flat-bottomed microtiter plates (TPP, Trasadingen, Switzerland) 0.2 × 10^6^ cells per well. Each sample was treated for 18 h or 5 days with 0, 1, 10 and 100 ng of DON (Sigma-Aldrich, St. Louis, MO, USA) per ml of culture medium in triplicate. After DON treatment, propidium iodide was added (0.2 µg) to each well for 3 min. The cells were then washed with DPBS. Afterwards, the proportion of viable cells from all events in lymphogate was analyzed with LSR Fortessa operated by Diva software (both BD Biosciences, Franklin Lakes, NJ, USA). Lymphogate was set as an area with the majority of lymphocytes based on forward and side scatter properties.

### 5.5. PBMC Proliferation Activity Assay

The proliferation activity of lymphocytes cultured with DON was determined using a mitogen-driven proliferation assay. Two hundred μL of the PBMC suspension (1 × 10^6^ cells/mL) was transferred into the wells of a 96-well flat-bottomed microtiter plate. DON was added to cultured cells in a concentration range of 0, 1, 10 and 100 ng/mL in triplicates. As a control, we used the PBMC suspension only. Cells were cultured for 18 h or 5 days at 37 °C in 5% CO_2_. After cultivation, cells were stimulated with mitogens ConA (Sigma-Aldrich, St. Louis, MO, USA), PHA (Remel, Lenexa, KS, USA) and PWM (Sigma-Aldrich, St. Louis, MO, USA) at concentrations of 10, 40 and 10 μg/mL, respectively. Cells without added mitogen served as control samples. All samples were run in triplicate. The microplates were incubated at 37 °C under 5% CO_2_ atmosphere for the next three days. Twenty hours before harvesting (FilterMateHarvestor, Packard Bioscience Instrument Company, Meriden, CT, USA), 50 μL of medium with 3H-thymidine (5 μCi/mL) was added. The incorporation of 3H-thymidine was analyzed by a microplate scintillation and luminescence counter (TopCount NXT™, Packard Bioscience Instrument Company, Meriden, CT, USA). The results were expressed in terms of stimulation indices, which were calculated as the ratio of CPM in stimulated samples versus CPM in non-stimulated controls.

### 5.6. Cytokine Gene Expression Analysis—RNA Preparation and Quantitative RT-PCR Analysis

PBMC suspensions were seeded in 24-well flat-bottomed tissue culture plates (TPP, Trasadingen, Switzerland). Sorted lymphocyte subpopulations were cultured in sterile polystyrene 5 mL tubes (BD Falcon, Franklin Lakes, NJ, USA). DON was added to PBMC in the concentration range of 0, 1, 10 and 100 ng/mL in triplicate and to sorted cells at 10 ng/mL in duplicate. Cells were cultured for 18 h (PBMC and sorted cells) or 5 days (PBMC only) at 37 °C in 5% CO_2_. Four hours before the end of the cultivation samples were stimulated with 15 nM phorbol-myristate-acetate (PMA; Sigma-Aldrich, St. Louis, MO, USA) and 1 μg/mL ionomycin (Sigma-Aldrich, St. Louis, MO, USA). Afterwards, the cells were centrifuged and the cell pellets were lysed in TRI Reagent (Sigma-Aldrich, St. Louis, MO, USA) and stored at −80 °C until RNA isolation. The RNA phase was obtained from the homogenate mixed with bromoanisole by separation in a refrigerated centrifuge. A column-based isolation (RNeasy Mini Kit, QiagenGmBH, Hilden, Germany) was applied to the RNA phase and the purified total RNA was eluted in 18 µL of RNeasy free water. RNA was subsequently reversely transcribed using M-MLV reverse transcriptase (200 U) (Invitrogen, Carlsbad, CA, USA) and oligo-dT primers in 20 µL reactions at 37 °C for 1.5 h and stored at −20 °C until used. Non-template controls containing elution water and reaction mixture were included.

Genes targeted in RT-PCR of PBMC included IL-1β, IL-2, IL-8, IL-17, IFNγ and TNFα. The gene expression of IL-8, IL-17, IFNγ and TNFα was analyzed in sorted lymphocyte subpopulations. Hypoxanthine phosphoribosyltransferase (HPRT) was used as a housekeeping reference gene [61]. Primers for IL-1β, IL-17, TNFα and HPRT were adopted from Pavlova et al. [62], Stepanova et al. [51], Volf et al. [63], and Zelnickova et al. [64], respectively. The remaining primers were designed using Primer3 software (all the primers are listed in Table 4). Gene expression was determined by quantitative PCR in triplicate reactions in 384 well plates. Plates were automatically filled using a Nanodrop II liquid dispenser (Innovadyne Technologies, Santa Rosa, CA, USA) following the manufacturer’s recommendations. PCR was performed using QuantiTect SYBR Green PCR Master Mix (Qiagen, Hilden, Germany) on a LightCycler 480 (Roche, Basel, Switzerland) under the following conditions: denaturation at 95 °C for 15 min and 45 amplification cycles at 95 °C for 15 s, 58 °C for 30 s and 72 °C for 30 s. The PCR reaction mixture consisted of 0.5 μL of cDNA, 1.5 μL of master mix and 10 pmol of each pair of primers (1 μL) (Generi Biotech, Czech Republic) in a final volume of 3 µL. A non-template control to test the assay reagents for contamination was included in each run. The relative gene expression of cytokine genes was calculated as multiples of the reference gene expression (i.e., *x*-fold value of reference gene) using the following formula: [1/(2^Ct target gene^)]/[1/(2^Ct HPRT^)].

### 5.7. Flow Cytometry Analysis of Cytokine Production in Lymphocyte Subpopulations after DON Exposure

Cell suspensions of PBMC were seeded in 96-well flat-bottomed tissue culture plates. DON (10 ng/mL) was added to cultured cells in the concentration in triplicate. Cells were cultured for 18 h at 37 °C in 5% CO_2_. Four hours before the end of the cultivation, samples were stimulated by 15 nM PMA and 1 μg/mL ionomycin with the addition of a protein transport inhibitor, brefeldinA (10 μg/mL, Sigma-Aldrich, St. Louis, MO, USA).

**Figure A2:**
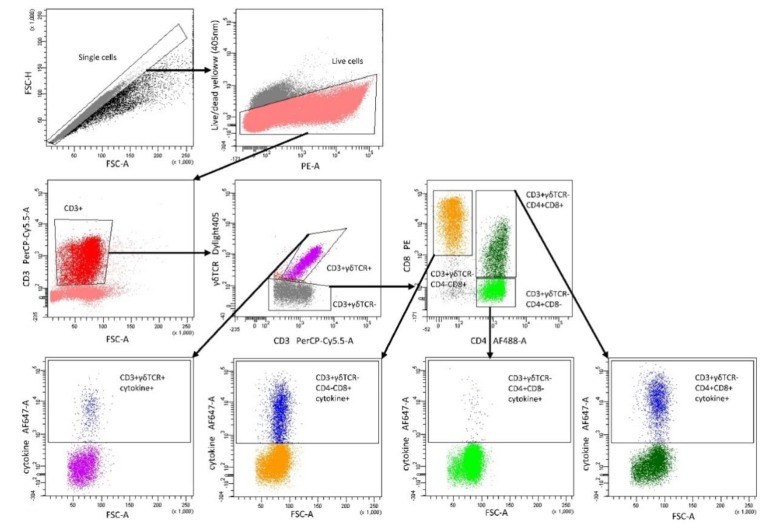
Gatting strategy—Flow cytometry analysis of cytokine production in lymphocyte subpopulations after DON exposure Doublets and dead cells identified by Live/dead probe (Fixable yellow dead cell stain kit for 405 nm excitation) were excluded. Subsequently, T cells subpopulations were defined from all CD3 positive cells and percentage of population positive for cytokine (IFNγ, IL-17 or TNFα) was evaluated. Dot plots show data from one representative sample where IFNγ was stained as cytokine.

Afterwards, stimulated PBMC were stained for surface markers. The following combinations of primary antibodies were used: CD3 (PerCP-Cy5.5-conjugated, BB23-8E6-8C8, BD Biosciences, Franklin Lakes, NJ, USA), CD4 (unconjugated, 10.2H2, IgG2b), γδTCR (unconjugated, PGBL22A, IgG1) and CD8α (unconjugated, 76-2-11). Fluorochrome-conjugated mouse isotype-specific goat antisera were used for unconjugated primary antibody visualization: Alexa Fluor 488 (anti-IgG2b, Invitrogen, Carlsbad, CA, USA), Dylight405 (anti-IgG1) and Phycoerythrin (anti-IgG2a, Invitrogen, Carlsbad, CA, USA). After surface marker staining the samples were fixed and permeabilized using an Intrastain kit (Dako, Glostrup, Denmark). During the permeabilization step, anti-IL-17 antibody (Alexa Fluor 647-conjugated, SCPL1362, BD Biosciences, Franklin Lakes, NJ, USA) oranti-IFN-gamma antibody (Alexa Fluor 647-conjugated, CC302, Bio Rad, Hercules, CA, USA) or anti-TNF-alpha antibody (APC-conjugated, MAb11, BD Biosciences, Franklin Lakes, NJ, USA) or the isotype control (mouse IgG1 negative control: Alexa Fluor 647, Bio Rad, Hercules, CA, USA) was added. Data were acquired on an LSR Fortessa flow cytometer operated by Diva software (both BD Biosciences, Franklin Lakes, NJ, USA). Dead cells were identified by Live/dead probe (Fixable yellow dead cell stain kit for 405 nm excitation). For the data analyses performed in Diva software, doublets and dead cells were excluded from analysis. Subsequently, T cell subpopulations were defined from all CD3 positive cells and analyzed for cytokine production. The gating strategy is available in Appendix A (Figure A2).

### 5.8. Statistical Analysis

The data were analyzed using a non-parametric paired Wilcoxon test. Values *p* < 0.05 were considered significant (* *p* ≤ 0.05, ** *p* ≤ 0.01, *** *p* ≤ 0.001). Outliers were excluded according to Grubbs’ test. All calculations were performed with GraphPad Prism version 3.03 software (GraphPad Software, San Diego, CA, USA).

## Figures and Tables

**Table 1:** Proliferation of mononuclear cells after 100 ng/mL, 10 ng/mL and 1 ng/mL DON exposure. Values are expressed as stimulation indices (ratios between counts per minute (CPM) of mitogen stimulated cells and CPM of non-stimulated cells) mean ± standard deviation. Data of PBMC samples are from nine pigs in triplicate. Statistically significant differences between DON exposed samples and control samples are marked with asterisks (* *p* ≤ 0.05; ** *p* ≤ 0.01).

Time of Stimulation	Mitogen	Control	DON 100 ng/mL	DON 10 ng/mL	DON 1 ng/mL
18 h	ConA	65.55 ± 23.47	39.31 ± 21.19 *	54.05 ± 37.55	50.63 ± 28.83
	PHA	9.77 ± 5.61	10.71 ± 5.78	5.69 ± 3.21	9.75 ± 8.32
	PWM	86.63 ± 39.08	47.47 ± 23.37 **	69.04 ± 42.59	69.67 ± 37.81
5 days	ConA	12.62 ± 11.31	8.74 ± 7.26	8.66 ± 10.51	8.93 ± 6.90
	PHA	7.60 ± 6.06	6.74 ± 6.30	6.34 ± 3.97	10.08 ± 5.85
	PWM	13.37 ± 8.83	8.27 ± 6.54 *	16.73 ± 15.84	18.19 ± 15.51

**Table 2:** Spontaneous proliferative activity of mononuclear cells after 100 ng/mL, 10 ng/mL and 1 ng/mL DON exposure. Values are expressed as CPM mean ± standard deviation. Data of PBMC samples from nine pigs in triplicate. Statistically significant differences between DON exposed samples and control samples are marked with asterisk (* *p* ≤ 0.05).

Time of Stimulation	Control	DON 100 ng/mL	DON 10 ng/mL	DON 1 ng/mL
18 h	52.33 ± 18.4	73.11 ± 27.93 *	56.25 ± 16.22	56.75 ± 12.06
5 days	47.44 ± 17.88	48.44 ± 19.15	51.22 ± 17.54	48.56 ± 18.90

**Table 3:** Relative expression of cytokine mRNA in PBMC after 100 ng/mL, 10 ng/mL and 1 ng/mL DON exposure. Values are expressed as mean of multiples of the reference gene expression ± standard deviation. Data of PBMC samples from nine pigs are in triplicate. Statistically significant differences between DON exposed samples and control samples are marked with asterisks (* *p* ≤ 0.05, ** *p* ≤ 0.01, *** *p* ≤ 0.001, NS (non significant) *p* > 0.05).

Time of Exposure	Cytokine	Relative Expression of Cytokine mRNA										
		DON Concentration (Mean ± SD)										
		Control 0 ng/mL		100 ng/mL			10 ng/mL			1 ng/mL		
		Mean	SD	Mean	SD	*p* Value	Mean	SD	*p* Value	Mean	SD	*p* Value
**18 h**	IL-1β	2.28	±1.23	3.14	±1.608	*	2.77	±1.85	NS	1.83	±1.20	NS
	IL-2	56.52	±16.77	49.76	±8.89	NS	45.14	±21.23	*	35.94	±20.87	**
	IL-8	9.56	±4.51	13.18	±6.40	**	7.98	±3.02	NS	7.17	±3.52	NS
	IL-17	3.41	±1.73	3.11	±1.25	NS	2.87	±1.51	*	2.62	±1.29	**
	IFNγ	7.97	±3.44	7.47	±2.43	NS	7.01	±2.58	**	6.96	±2.35	NS
	TNFα	1.78	±0.78	1.87	±0.68	NS	1.11	±0.53	**	1.11	±0.62	**
**5 days**	IL-1β	2.53	±2.91	0.98	±0.71	**	1.12	±1.1	**	1.28	±0.95	**
	IL-2	49.22	±25.73	27.17	±12.1	***	35.10	±21.5	**	37.21	±17.11	NS
	IL-8	13.05	±12.38	16.37	±15.55	NS	9.68	±9.05	*	9.37	±6.69	*
	IL-17	2.48	±1.81	1.30	±0.56	***	1.54	±0.63	*	1.41	±0.6	*
	IFNγ	9.60	±3.57	6.10	±2.11	***	9.08	±3.59	NS	8.98	±4.56	*
	TNFα	2.34	±1.66	1.82	±1.26	NS	1.47	±0.95	**	1.69	±1.18	**

**Table 4:** Primers.

Gene	Forward 5′-3′	Reverse 5′-3′
HPRT	GAGCTACTGTAATGACCAGTCAACG	CCAGTGTCAATTATATCTTCAACAATCAA
IFNγ	TGCAGATCCAGCGCAAAGCCATCAG	TTGATGCTCTCTGGCCTTGGAACATAGTC
IL-1β	GGGACTTGAAGAGAGAAGTGG	CTTTCCCTTGATCCCTAAGGT
IL-8	TGAAGAGAACTGAGAAGCAACAACAACAGCAG	TCTTGGGAGCCACGGAGAATGGGT
IL-17	ACATGCTGAGGGAAGTTCTTGTC	ATCCTCGTCCCTGTCACTGC
TNFα	CCCCCAGAAGGAAGAGTTTC	CGGGCTTATCTGAGGTTTGA
